# Network Coding-Enhanced Polar Codes for Relay-Assisted Visible Light Communication Systems

**DOI:** 10.3390/e26121112

**Published:** 2024-12-19

**Authors:** Congduan Li, Mingyang Zhong, Yiqian Zhang, Dan Song, Nanfeng Zhang, Jingfeng Yang

**Affiliations:** 1 School of Electronics and Communication Engineering, Sun Yat-sen University, Shenzhen 518107, China; licongd@mail.sysu.edu.cn (C.L.); jobzmy@163.com (M.Z.); songd8@mail2.sysu.edu.cn (D.S.); 2 Shenzhen Key Laboratory of Navigation and Communication Integration, Shenzhen 518107, China; 3 Guangdong Provincial Key Laboratory of Intelligent Port Security Inspection, Huangpu Customs District P.R. China, Guangzhou 510700, China; nf_zhang@126.com; 4 Guangzhou Institute of Industrial Intelligence, Guangzhou 511458, China; yangjf@gz.sia.cn

**Keywords:** visible light communication, polar codes, physical-layer network coding

## Abstract

This paper proposes a novel polar coding scheme tailored for indoor visible light communication (VLC) systems. Simulation results demonstrate a significant reduction in bit error rate (BER) compared to uncoded transmission, with a coding gain of at least 5 dB. Furthermore, the reliable communication area of the VLC system is substantially extended. Building on this foundation, this study explores the joint design of polar codes and physical-layer network coding (PNC) for VLC systems. Simulation results illustrate that the BER of our scheme closely approaches that of the conventional VLC relay scheme. Moreover, our approach doubles the throughput, cuts equipment expenses in half, and boosts effective bit rates per unit time-slot twofold. This proposed design noticeably advances the performance of VLC systems and is particularly well-suited for scenarios with low-latency demands.

## 1. Introduction

The rapid flickering capability of light-emitting diodes (LEDs) allows visible light communication (VLC) technology to offer the simultaneous provision of both lighting and telecommunications. This offers abundant spectrum resources, improves environmental management, enhances energy efficiency, and is immune to electromagnetic interference [1]. As a result, since its emergence in the 21st century, VLC has risen as a crucial focus in wireless communication research. VLC, leveraging the unlicensed ultrawide bandwidth of visible light, offers high-speed data transmission, minimal latency, and integration with ubiquitous energy-efficient LED lighting. It is highlighted as a key enabler for next-generation networks like 6G, with applications spanning IoT, healthcare, augmented reality, and smart transportation. The technology’s inherent advantages, including immunity to electromagnetic interference, low energy consumption, and environmental sustainability, make it particularly suited for indoor and densely populated environments. The future of VLC also holds significant potential through the integration of Lighting, Sensing, and Communication (LiSAC) systems [2]. VLC techniques have been applied in various scenarios; for instance, ref. [3] investigates the enhancement of Federated Learning (FL) through a hybrid system that integrates VLC and Radio Frequency (RF). By leveraging VLC to mitigate RF bandwidth constraints and adopting model compression techniques to reduce communication overhead, the study develops an optimization framework for user selection and bandwidth allocation, aiming to minimize FL training loss. In [4], a system is proposed that integrates VLC, visible light positioning (VLP), and flicker-mitigation illumination, all while ensuring physical-layer security. By leveraging polar codes, the system achieves secure and reliable VLC transmission coupled with high-precision indoor positioning. The capacity region of multiple access channels in VLC networks under both peak and average optical power constraints is investigated in [5]. The authors propose new inner and outer bounds for the channel capacity by developing an innovative approach using an inexact gradient descent method to handle the complex optimization problem. By considering the unique characteristics of VLC, such as non-negative real-valued signals and power constraints, the study demonstrates that the optimal input distributions are discrete and vary depending on the signal-to-noise ratio (SNR). However, VLC is susceptible to interference caused by power attenuation and inherent noise, leading to error propagation. To maintain high-quality telecommunication while controlling costs, the adoption of channel coding is essential. Channel codes are designed to enhance the reliability of data transmission over noisy communication channels by adding redundancy to the transmitted data. This redundancy enables the receiver to detect and, in some cases, correct errors that may occur during transmission. Among the various coding techniques, polar codes are promising [6]. Channel polarization involves combining and splitting channels in a recursive manner, leading to a polarization effect, where the symmetric capacity terms of the resulting channels tend towards 0 or 1. Polar codes, constructed based on this idea, can achieve the symmetric capacity of any given binary-input memoryless channel. In addition, polar codes offer significant advantages, such as low computational complexity, the ease of implementation, and flicker-free operation [7], making them highly suitable for integration into VLC systems. In this study, we propose a novel scheme that incorporates polar codes into VLC channels. The scheme employs a Gaussian approximation to model the polarization behavior within the VLC channel and develops a specialized class of polar codes optimized for VLC. Additionally, a tailored decoding algorithm is designed to accommodate the specific characteristics of the VLC channel. Our experimental results demonstrate that the proposed scheme achieves a minimum coding gain of 5 dB, along with a substantial reduction in bit error rate (BER), significantly improving the reliability of VLC systems. This advancement expands the application of polar codes and enhances the overall performance of VLC technology.

The transmission efficiency metric is a crucial metric investigated in the field of telecommunications. VLC, owing to its short wavelength and restricted diffraction, demonstrates susceptibility to obstruction and interference in the transmission link, giving rise to the following scenarios:

- The transmitter and receiver are not in a direct line-of-sight communication;
- The transmitter and receiver are situated at a considerable distance, making reliable communication challenging.

Therefore, implementing a relay scheme is essential to enable bidirectional VLC among multiple users. In traditional relay schemes, relay nodes simply store and forward the data received from source nodes. However, simply routing or replicating at intermediate nodes is generally not optimal. Unlike traditional methods that treat information as a “fluid” that can simply be routed or replicated, network coding [8] employs coding strategies at intermediate nodes to optimize information flow. This approach has been shown to be beneficial in saving bandwidth and increasing throughput in multicast scenarios. By allowing nodes to process and combine information in innovative ways, network coding facilitates the more efficient use of network resources [9]. Considering physical-layer network coding (PNC) in a relay-assisted communication system, ref. [10] introduces the PNC concept and its application in improving network throughput. Compared with traditional transmission and straightforward network coding schemes in terms of BER performance, PNC can achieve higher throughput with a slightly worse BER in low-SNR conditions. PNC utilizes the transformation of mutual interference among wireless signals into beneficial information, thus improving the performance of VLC systems. Traditional VLC systems face significant BER degradation due to photodiode saturation caused by ambient light. The PNC–VLC system proposed in [11] mitigates this issue by utilizing two LEDs of the same color to transmit different data streams, allowing naturally overlapped signals at the receiver to form network-coded messages. This approach improves system robustness and stability under various ambient lighting conditions. A novel scheme combining polar coding and PNC is proposed for two-user downlink non-orthogonal multiple access (NOMA) systems [12]. By leveraging PNC, the scheme efficiently manages inter-user interference by transmitting a superimposed XORed message of both users and the weaker user’s message. By exploiting the polarization effect and PNC principle, polar decoding can directly recover the individual user messages, achieving user fairness and maximizing effective throughput.

Recent years have seen significant progress in applying network coding and polar codes to VLC technology. S. Song presents an experimental study on PNC based on precoded Orthogonal Frequency Division Multiplexing (OFDM) in relay-assisted VLC [13]. It demonstrates the application of PNC in a 2.16 Gbit/s asymmetric two-way-relay VLC network, showcasing significant performance improvements and enabling full-duplex transmission. The study focuses on the use of precoded OFDM to address the bandwidth limitation issue in VLC systems. Y. Hong introduces a full-duplex channel-aware physical-layer network coding scheme for relay-assisted OFDM-based VLC networks [14]. The proposed scheme aims to boost the capacity and throughput of VLC networks by addressing the high-frequency power fading issue. S. Wen initiated an experimental research on polar codes constructed by Gaussian Approximation (GA) for VLC systems [15]. This work investigated the impact of key parameters in the coding and decoding scheme through numerical simulations. The research demonstrates that polar codes, with their low decoding time and space complexity, are well-suited for VLC systems. O. P. Babalola proposed an efficient channel coding scheme for dimmable VLC systems [7]. This work discusses the integration of polar codes with Knuth’s balancing code to enhance the performance of VLC. The proposed scheme aims to address the challenges of dimming and flicker-free operation in VLC. H. Hematkhah evaluated the performance of polar channel coding on a practical VLC link, comparing it with Turbo and LDPC codes [16]. The study was conducted in terms of BER versus SNR. The results indicate that polar codes outperform Turbo and LDPC codes on VLC-based applications. B. Sun explored the integration of LDPC and PNC for two-way relay channels, with particular attention on the complexities introduced by varying frequency offsets [17]. This work investigated the impact of these frequency offsets on the performance of the communication system and proposed techniques to mitigate their adverse effects, ensuring reliable communication. Additionally, J. Du proposed a low-decoding complexity scheme for two-user Gaussian multiple-access channels (GMACs) using LDPC codes aided by PNC [18]. Numerical results show that the proposed scheme has better BER performance compared to existing LDPC-coded schemes for GMACs.

Current research primarily focuses on combining LDPC with PNC, with limited attention given to polar codes. However, polar codes have gained significant recognition for their capacity-achieving performance and error-correction capabilities under successive cancellation decoding, making them highly suitable for emerging communication systems. Building upon previous work, this study introduces a novel method that integrates PNC with polar codes, specifically in the context of VLC systems. While LDPC-based methods have shown promise, they often suffer from higher decoding complexity and latency, which are critical drawbacks in low-latency and resource-constrained VLC applications. In addition, network coding, particularly PNC, improves system throughput and minimizes communication latency, making it ideal for real-time applications. By combining PNC with polar codes, this study leverages the error correction and stability benefits of polar codes alongside the efficiency and reduced transmission time enabled by network coding. The proposed method thus addresses key challenges in VLC systems, such as achieving high throughput, low latency, and cost-effectiveness, which are crucial for practical deployment. Experimental results indicate that the BER of the proposed method closely aligns with that of traditional schemes, yet it achieves superior throughput, reduced equipment requirements, and enhanced cost-efficiency. By integrating polar codes with PNC, this study provides a comprehensive framework that not only enhances communication reliability but also ensures the efficient use of network resources.

The structure of this paper is as follows. Section 2 provides an overview of the foundational concepts, including the VLC system, polar codes, network coding, and relay strategies tailored for VLC. Section 3 focuses on the joint implementation of polar codes and PNC in the VLC framework. Section 4 highlights the simulation results of the proposed approach, and Section 5 offers concluding remarks.

## 2. Preliminaries

In this section, we give a brief of the fundamental principles and framework of the adopted algorithm, highlighting four key aspects: visible light communication systems, polar codes, network coding, and relay schemes.

### 2.1. Visible Light Communication Systems

VLC, a technology that concurrently addresses indoor lighting and data exchange, is inherently energy-efficient. It holds substantial potential for application in wireless communication.

Indoor VLC systems commonly employ Intensity Modulation Direct Detection (IM/DD) technology, a choice made for its cost-effectiveness and simplicity [19]. The mathematical representation of the associated channel is

(1) y=hx+n,

where *h* represents the instantaneous gain of the VLC channel, *n* denotes the signal-independent noise, *x* signifies the transmitted sequence, and *y* indicates the received sequence.

Considering the existence of a line of sight (LOS) between the transmitter and receiver, there are two types of VLC channels: the LOS model and the non-line of sight (NLOS) model [20]. Furthermore, it is important to note that signal transmission is inevitably influenced by indoor factors like room dimensions, the reflectivity of walls, and the directivity characteristics of the transmitting and receiving devices. For the sake of practicality in our experiment, we opt for the NLOS model in simulations. The VLC NLOS channel model includes not only direct signals but also those indirectly reflected by the walls. The room’s dimensions are 5 m long, 5 m wide, and 3 m high. Both the transmitter and receiver are centrally positioned within the room, with a communication distance of 2.15 m between them. The transmitter has a semi-angle of 70 degrees, whereas the receiver’s field of view spans 60 degrees. The physical area of the photodetector measures 1 square centimeter, and the surrounding walls possess a reflection coefficient of 0.8.

Regarding the NLOS model, the received light power is

(2) $$P_{r}=\left(H_{LOS}+H_{NLOS}\right)P_{t}=\left(H_{LOS}+\sum_{refl}H_{refl}\right)P_{t}\;.$$

In this context, $H_{LOS}$ and $H_{NLOS}$ denote the transfer functions for the direct and non-direct links, respectively. The transfer function of the reflected link is represented by $H_{refl}$. Additionally, $P_{t}$ indicates the power of the transmitted light, while $P_{r}$ refers to the power of the received light.

**Figure 1:**
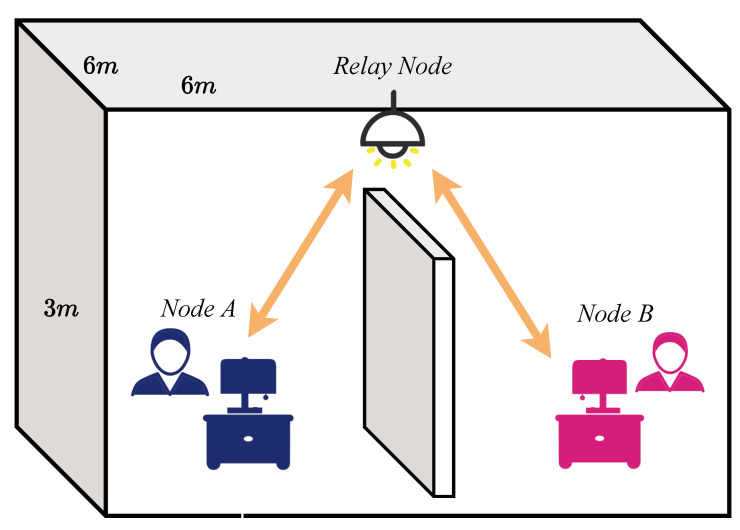
The relay-assisted VLC systems model.

As shown in Figure 1, when node A endeavors to transmit data to node B, a node that lies beyond the reach of direct communication, the utilization of a relay node becomes crucial for maintaining smooth communication between them, leading to the relay-assisted VLC system model. The dimensions of the studied model are 6 m in length, 6 m in width, and 3 m in height. The relay node *R*, located at the coordinates (3,3,3), is positioned centrally within the space. Nodes *A* and *B*, symmetrically placed relative to the relay node, are mounted on the ceiling. Their exact coordinates are (1.5,1.5,0.85) and (4.5,4.5,0.85), respectively. 

### 2.2. Polar Codes

Polar codes, belonging to the category of Forward Error Correction (FEC) codes, are founded upon the theory of channel polarization. This theory involves the processing of channels, specifically channel combining and splitting, to select reliable channels with capacities nearing 1 for the transmission of information bits. Conversely, the less reliable channels, with capacities closer to 0, are designated for the transmission of “frozen” bits, which are uniformly set to zero [21].

Let $u_{1}^{N}$ denote the source sequence, consisting of $u_{1},u_{2},\cdots ,u_{N}$ bits. Among these *N* bits, *K* are information bits, while the remainder are “frozen” bits. The positions of both the information and “frozen” bits are predefined and known to both the transmitter and receiver. The code rate is given by R=K/N. Following the polar encoding process, the codeword $x_{1}^{N}=\left(x_{1},x_{2},\cdots ,x_{N}\right)$ is produced as

(3) $$x_{1}^{N}=u_{1}^{N}G_{N}=u_{1}^{N}B_{N}F^{⊗n},F=\left[\begin{array}{cc} 1 & 0\\ 1 & 1 \end{array}\right],$$

where $G_{N}$ represents the generation matrix, $B_{N}$ represents the bit reversal matrix, and ⊗ denotes the Kronecker Product.

In [22], a scheme employing polar codes tailored for the VLC channel is introduced. This scheme demonstrated a notable reduction in errors attributed to noise interference during light signal transmission. It led to an improvement in coding gain and an expansion of the reliable communication coverage. The proper selection of parameters, such as code length and rate, can further lower the BER of the VLC system. Thus, to enhance the reliability of the VLC system, we proceed with the polar code construction method described in the previously mentioned scheme. Furthermore, the decoding process is carried out using the successive cancellation algorithm.

### 2.3. Network Coding

Traditionally, relay nodes typically forward information directly from source nodes to destination nodes, without any processing. However, with network coding, relay nodes encode messages and send the encoded information to the destination. This enables the network to achieve its maximum theoretical transmission capacity, as stated by the max-flow min-cut theorem.

The PNC concept, first presented in [10], builds upon the idea of network coding but applies it to the physical layer. Unlike traditional relay nodes, PNC-enabled relays do not decode signals individually; they instead process the combined signals directly through mapping and modulation, which achieves network coding. This approach leads to a decrease in transmission time slots and an increase in throughput rates.

### 2.4. Relay Schemes

**Figure 2:**
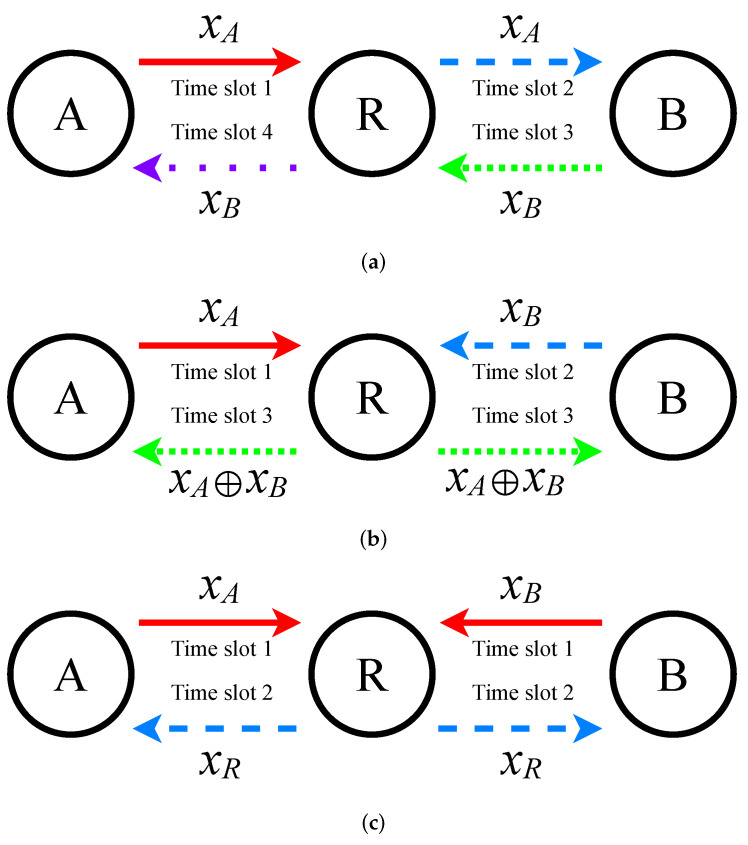
Comparison of different relay schemes. (**a**) The traditional relay scheme. (**b**). The relay scheme employs direct network coding (DNC). (**c**) The model of the physical-layer network coding (PNC) relay scheme.

Wireless communication systems are prone to mutual interference, which occurs when electromagnetic waves operating on nearby frequencies disrupt each other. This interference can ultimately degrade both accuracy and overall performance [23]. When signals share the same channel, one signal can act as interference for another. This is the reason why, in the conventional relaying scheme, only one node is authorized to transmit data during a specific time slot [24]. This is depicted in the schematic diagram in Figure 2a:

- In the first time slot, Node A transmits a signal $x_{A}$ to the relay node;
- During the second time slot, the relay node receives the signal $x_{A}$ from Node A and then relays it to Node B;
- In the third time slot, Node B sends a signal $x_{B}$ to the relay node;
- Finally, in the fourth time slot, the relay node forwards the signal $x_{B}$ that it received from Node B to Node A.

To enable an exchange of information between A and B, four time slots are required, leading to an information transmission efficiency of 1/4.

Shown in Figure 2b is a schematic diagram illustrating the Direct Network Coding (DNC) relay scheme:

- During the first time slot, Node A transmits a signal $x_{A}$ to the relay node;
- In the second time slot, Node B transmits a separate signal $x_{B}$ to the relay node;
- In the third time slot, the relay node applies an XOR operation to the two received signals ($x_{A}⊕x_{B}$) and broadcasts the resultant signal to both A and B simultaneously.

To avoid mutual interference, A and B transmit their signals in separate time slots. Once receiving the signals, the relay node employs DNC, often in the form of an XOR operation, as an encoding process [25]. The relay node then simultaneously broadcasts the encoded results to both A and B. Subsequently, node A retrieves the desired information by performing an XOR operation between the received signal and its original signal, resulting in $x_{A}⊕x_{B}⊕x_{A}=x_{B}$. Node B follows an identical procedure. This approach requires a total of 3 time slots for A and B to successfully exchange information, resulting in an information transmission efficiency of 1/3. In comparison to the conventional relay scheme, this approach enhances the VLC system’s throughput by 33%.

In contrast to the aforementioned relay schemes, which treat simultaneous channel signals as interference to be avoided, the PNC relay mode transforms interference into useful information, thereby enhancing the performance of the VLC system. The schematic diagram of the PNC relay scheme is illustrated in Figure 2c:

- During the first time slot, A and B transmit their respective signals, $x_{A}$ and $x_{B}$, to the relay node simultaneously;
- During the second time slot, the relay node processes the combined signal by applying a predefined mapping method, which effectively encodes the signals. The relay then simultaneously broadcasts this encoded signal to both A and B.

The PNC relay scheme reduces the time required by one slot, resulting in a 50% increase in throughput compared to the DNC relay scheme. It also saves two time slots, effectively doubling the throughput when compared to the traditional relay scheme. In summary, the PNC relay scheme considerably enhances the throughput.

## 3. Theoretical Analysis

We present a scheme that utilizes polar codes specifically designed for the VLC channel. Additionally, we detail a model that integrates polar codes and network coding, including PNC in a relay-assisted VLC system. We also conduct a thorough analysis of the theoretical performance of this scheme.

### 3.1. Polar Codes for VLC Channels

#### 3.1.1. Encoding

The fundamental principle of channel polarization centers on optimally selecting information bits. While binary erasure channels (BECs) offer relatively straightforward analysis, evaluating capacity for complex non-BECs remains a significant research challenge. VLC channels exemplify this complexity, presenting substantial obstacles in accurately assessing polarization reliability. Our research introduces an innovative approach to evaluating the reliability of the polarization channel, along with a thorough analysis of the relevant polarization phenomenon.

In a VLC IM/DD system, the On–Off Keying (OOK) modulation technique enables a probabilistic characterization of channel output. The conditional probability of a channel producing an output *y* when the transmitted bit is either 0 or 1 is described by a normal distribution, also known as a Gaussian distribution, and is expressed as follows:

(4) $$\begin{array}{c} P\left(y|x=0\right)=\frac{1}{\sqrt{2\pi \sigma^{2}}}\exp\left(-\frac{y^{2}}{2\sigma^{2}}\right), \end{array}$$

(5) $$\begin{array}{c} P\left(y|x=1\right)=\frac{1}{\sqrt{2\pi \sigma^{2}}}\exp\left(-\frac{{\left(y-h\right)}^{2}}{2\sigma^{2}}\right). \end{array}$$

The Bhattacharyya parameter is a key metric used in the design and analysis of polar codes to evaluate the reliability of a channel. It provides a way to measure how distinguishable the transmitted signal is from the noise and is closely related to the channel error probability [6]. For a binary-input channel W(y|x) with input x∈{0,1} and output *y*, the Bhattacharyya parameter Z(W) is defined as

(6) $$Z\left(W\right)=\sum_{y\in Y}\sqrt{W(y|0)W(y|1)}$$

where Y is the set of possible outputs of the channel. The parameter Z(W) lies in the range [0,1]. If Z(W)=0, the channel is perfectly reliable, as the outputs for x=0 and x=1 are completely distinguishable. If Z(W)=1, the channel is completely unreliable, as the outputs for x=0 and x=1 are indistinguishable. By the definition of the Bhattacharyya parameter, its mathematical expression in a VLC channel is given by

(7) $$\begin{array}{cc} Z(W) & =\int_{-\infty }^{\infty }\sqrt{P(y|0)P(y|1)}dy\\ \; & =\int_{-\infty }^{\infty }\sqrt{\frac{1}{\sqrt{2\pi \sigma^{2}}}e^{-\frac{y^{2}}{2\sigma^{2}}}\frac{1}{\sqrt{2\pi \sigma^{2}}}e^{-\frac{{\left(y-h\right)}^{2}}{2\sigma^{2}}}}dy\\ \; & =e^{-\frac{h^{2}}{8\sigma^{2}}}. \end{array}$$

In the context of VLC channel modeling, as shown in Equation (1), the log-likelihood ratio (LLR) for signal outputs demonstrates a near-Gaussian probabilistic behavior. The signal transformations of sub-channel $W_{N}^{\left(i\right)}$ can be characterized through recursive computational approaches, where each channel’s logarithmic likelihood representation $L_{N}^{\left(i\right)}$ exhibits a distinctive mean value $m_{N}^{\left(i\right)}$. Based on a Gaussian approximation principle, these mean values emerge from the following recursive mathematical formulations [26]:

(8) $$\begin{array}{c} m_{N}^{\left(2i\right)}=2m_{N/2}^{\left(i\right)}, \end{array}$$

(9) $$\begin{array}{c} m_{N}^{\left(2i-1\right)}=\phi^{-1}\left(1-{\left(1-\phi \left(m_{N/2}^{\left(i\right)}\right)\right)}^{2}\right). \end{array}$$

The computational complexity of the function ϕ(x) is prohibitively high, making it challenging to solve directly. To address this, researchers have proposed an approximate calculation formula, referred to as Approximate GA [27]. The function ϕ(x) in this context is expressed as follows:

(10) $$\phi \left(x\right)=\left\{\begin{array}{cc} \exp\left(-0.4527x^{0.86}+0.0218\right), & 0<x<10\\ \sqrt{\frac{\pi }{x}}\left(1-\frac{10}{7x}\right)\exp\left(-\frac{x}{4}\right), & 10\leq x. \end{array}\right.$$

The initial value $m_{1}^{\left(1\right)}$ is determined using Equation (7), while the remaining mean values $m_{N}^{\left(i\right)}$ are computed iteratively based on Equations (8) and (9). The noise variance for the *i*-th sub-channel, denoted as ${\left(\sigma_{N}^{\left(i\right)}\right)}^{2}$, is related to the mean value $m_{N}^{\left(i\right)}$ as follows:

(11) $${\left(\sigma_{N}^{\left(i\right)}\right)}^{2}=\frac{2}{m_{N}^{\left(i\right)}}.$$

Based on Equations (7) and (11), the Bhattacharyya parameter for the VLC channel is expressed as

(12) $$Z\left(W_{N}^{\left(i\right)}\right)=\exp\left(-\frac{h^{2}}{8{\left(\sigma_{N}^{\left(i\right)}\right)}^{2}}\right)=\exp\left(\frac{-h^{2}m_{N}^{\left(i\right)}}{16}\right).$$

By assessing the Bhattacharyya parameter for each sub-channel $W_{N}^{\left(i\right)}$, we pinpoint the *K* sub-channels that exhibit the smallest values of $Z\left(W_{N}^{\left(i\right)}\right)$. The indices corresponding to these chosen sub-channels constitute the set *A*, which represents the information bits. This methodology underpins the polar encoding strategy tailored for the VLC channel.

#### 3.1.2. Decoding

The decoding approach for a VLC channel operates as described. Consider the input to the polarized sub-channel as $u_{i}$ and the output comprising the received sequence $\left(y_{1}^{N}\right)$ along with the preceding i−1 bits denoted as $u_{1}^{i-1}$. To initiate, the LLR for a VLC channel employing OOK modulation is determined as follows. In the case where N=1, the LLR, designated as $L_{1}^{\left(1\right)}\left(y_{i}\right)$, for the symbol $y_{i}$ outputted by the *i*-th sub-channel is calculated.

(13) $$\begin{array}{cc} L_{1}^{\left(1\right)}\left(y_{i}\right) & =\ln\frac{W\left(y_{i}|0\right)}{W\left(y_{i}|1\right)}\\ \; & =\ln\frac{\frac{1}{\sqrt{2\pi \sigma^{2}}}\exp\left(-\frac{y_{i}^{2}}{2\sigma^{2}}\right)}{\frac{1}{\sqrt{2\pi \sigma^{2}}}\exp\left(-\frac{{\left(y_{i}-h\right)}^{2}}{2\sigma^{2}}\right)}\\ \; & =\frac{h^{2}-2hy_{i}}{2\sigma^{2}}, \end{array}$$

where $W\left(y_{i}|x_{i}=0\right)$ and $W\left(y_{i}|x_{i}=1\right)$ denote the subchannel transition probabilities. For the bit $u_{i}$ of the information sequence, where *i* belongs to the set *A*, the LLR is determined using the received sequence $y_{1}^{N}$ along with the bits ${\hat{u}}_{1}^{i-1}$ that have been previously estimated.

(14) $$L_{N}^{\left(i\right)}\left(y_{1}^{N},{\hat{u}}_{1}^{i-1}\right)=\ln\frac{W_{N}^{\left(i\right)}\left(y_{1}^{N},{\hat{u}}_{1}^{i-1}|0\right)}{W_{N}^{\left(i\right)}\left(y_{1}^{N},{\hat{u}}_{1}^{i-1}|1\right)},$$

(15) $$\begin{array}{cc} L_{N}^{\left(2i-1\right)}\left(y_{1}^{N},{\hat{u}}_{1}^{2i-2}\right)= & L_{N/2}^{\left(i\right)}\left(y_{1}^{N/2},{\hat{u}}_{1,o}^{2i-2}⊕{\hat{u}}_{1,e}^{2i-2}\right)\\ & ⊙L_{N/2}^{\left(i\right)}\left(y_{N/2+1}^{N},{\hat{u}}_{1,e}^{2i-2}\right), \end{array}$$

(16) $$\begin{array}{cc} L_{N}^{\left(2i\right)}\left(y_{1}^{N},{\hat{u}}_{1}^{2i-1}\right)= & \left(1-2{\hat{u}}_{2i-1}\right)L_{N/2}^{\left(i\right)}\left(y_{1}^{N/2},{\hat{u}}_{1,o}^{2i-2}⊕{\hat{u}}_{1,e}^{2i-2}\right)\\ & +L_{N/2}^{\left(i\right)}\left(y_{N/2+1}^{N},{\hat{u}}_{1,e}^{2i-2}\right), \end{array}$$

where the elements of ${\hat{u}}_{1}^{2i-2}$ are categorized into odd-indexed and even-indexed groups, represented by ${\hat{u}}_{1,o}^{2i-2}$ and ${\hat{u}}_{1,e}^{2i-2}$ respectively. The operation ⊕ is the modulo-2 addition operation, also referred to as binary addition. It is performed on binary numbers (i.e., numbers consisting of only 0s and 1s) and follows the rules of arithmetic in modulo 2. The operation ⊙ [6] is denoted as

(17) $$a⊙b=\log\left(\frac{1+e^{a+b}}{e^{a}+e^{b}}\right).$$

Using Equations (15) and (16), the computation of the LLR for a received sequence of length *N* can be reduced to N/2, with the recursive process terminating when *N* reaches 1. Subsequently, the decoded output sequence is generated by applying the following formula to the received sequence:

(18) $${\hat{u}}_{i}=\left\{\begin{array}{cc} 1, & L_{N}^{\left(i\right)}\left(y_{1}^{N},{\hat{u}}_{1}^{i-1}\right)<0\\ 0, & L_{N}^{\left(i\right)}\left(y_{1}^{N},{\hat{u}}_{1}^{i-1}\right)\geq 0. \end{array}\right.$$

The proposed polar code scheme serves to correct errors and bolster the anti-interference capabilities of VLC signals. Subsequent research will concentrate on integrating the prior findings with network coding technology, aiming to elevate the information transmission rate of the VLC system and further enhance its overall performance.

### 3.2. The Integration of Polar Codes and Direct Network Coding in a Relay-Assisted VLC System

**Figure 3:**
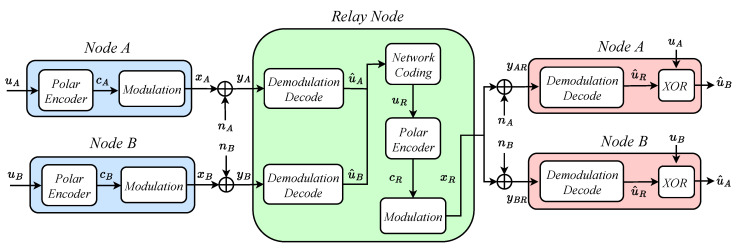
A model that integrates polar codes with direct network coding in a relay-assisted VLC.

The system model for combining polar codes with DNC is shown in Figure 3. The steps of this scheme are as follows:

- Nodes A and B utilize a polar encoder to encode their respective original information, denoted as $u_{A}$ and $u_{B}$. These encoded data are modulated and transmitted to the relay node, where they are received in two distinct time slots, potentially impacted by noise;
- After receiving $y_{A}$ and $y_{B}$, the relay node proceeds to estimate the original information through demodulation and decoding processes. Subsequently, it employs the DNC technique to encode the estimated results, ${\hat{u}}_{A}$ and ${\hat{u}}_{B}$, and simultaneously transmits these encoded outcomes to both nodes;
- Node A initially demodulates and decodes the relay’s transmission, $x_{R}$. Subsequently, utilizing its own original data, it performs a decoding operation on ${\hat{u}}_{R}$ to extract node B’s message, through the operation ${\hat{u}}_{R}⊕u_{A}=u_{B}$. Node B follows a similar process to retrieve node A’s message. This enables both nodes to successfully exchange information.

In a scheme that integrates polar codes with direct network coding, the VLC system requires dual equipment setups for demodulation and decoding at the relay node to handle signals from both source nodes simultaneously within a single time slot. This increases both device costs and computational expenses. To address these issues, this paper proposes a hybrid scheme that integrates polar codes with PNC.

### 3.3. Integration of Polar Codes and Direct Network Coding in Relay-Assisted VLC Systems

**Figure 4:**
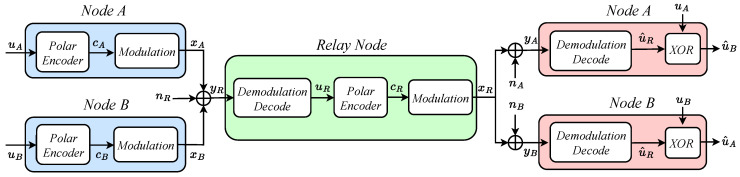
A model that integrates polar codes with physical-layer network coding in a relay-assisted VLC system.

Figure 4 showcases a system model that integrates polar codes with PNC. While the encoding process for this scheme shares similarities with the previous model, the following are the notable distinctions:

- During the same time slot, both A and B concurrently transmit their signals to the relay node;
- The relay node processes the superimposed signal using a distinct physical-layer mapping technique. It directly applies network coding to the combined signal, rather than treating the signals from nodes A and B as separate entities.

Here are the steps:

*(1).* The source nodes encode the original information $u_{A}$ and $u_{B}$ using polar encoders configured with the identical code rates, resulting in $c_{A}$ and $c_{B}$, respectively. These encoded signals are modulated to produce $x_{A}$ and $x_{B}$, which are transmitted simultaneously to the relay node. Upon passing through the VLC channel, the relay node receives a combined signal, represented as

(19) $$y_{R}=h\left(x_{A}+x_{B}\right)+n_{R},$$

where *h* denotes the VLC channel’s instantaneous gain, $y_{R}$ is the received signal, and $n_{R}$ is noise with a mean of zero and a variance of $\sigma^{2}$;

*(2).* The LLR of the combined signal $y_{R}$ is computed to directly derive the network-coded result for $u_{A}$ and $u_{B}$.

The mapping scheme at the relay node R is shown in Table 1, where $P_{t}$ and $P_{r}$ represent the transmitted light power and received light power. The relation between $P_{t}$ and $P_{r}$ is detailed in Equation (2). The XOR results of the two VLC signals can be obtained through the mapping scheme.

In particular, the LLR of $y_{R}$ is computed using the following formula:

(20) $$L\left(y_{R}^{i}\right)=L\left(c_{A}^{i}⊕c_{B}^{i}\right)=\ln\frac{P\left(c_{A}^{i}⊕c_{B}^{i}=0|y_{R}^{i}\right)}{P\left(c_{A}^{i}⊕c_{B}^{i}=1|y_{R}^{i}\right)},$$

where *i* epresents the index in the sequence.

When $c_{A}^{i}⊕c_{B}^{i}=0$, two possible scenarios arise:

(21) $$c_{A}^{i}⊕c_{B}^{i}=0\Rightarrow \left\{\begin{array}{c} c_{A}^{i}=0,c_{B}^{i}=0\Rightarrow x_{A}^{i}+x_{B}^{i}=0,\\ c_{A}^{i}=1,c_{B}^{i}=1\Rightarrow x_{A}^{i}+x_{B}^{i}=2. \end{array}\right.$$

When $c_{A}^{i}⊕c_{B}^{i}=1$, different corresponding scenarios emerge:

(22) $$c_{A}^{i}⊕c_{B}^{i}=1\Rightarrow \left\{\begin{array}{c} c_{A}^{i}=0,c_{B}^{i}=1\Rightarrow x_{A}^{i}+x_{B}^{i}=1,\\ c_{A}^{i}=1,c_{B}^{i}=0\Rightarrow x_{A}^{i}+x_{B}^{i}=1. \end{array}\right.$$

The prior probability of each situation is

(23) $$P\left(x_{A}^{i}+x_{B}^{i}=0\right)=\frac{1}{4},$$

(24) $$P\left(x_{A}^{i}+x_{B}^{i}=1\right)=\frac{1}{2},$$

(25) $$P\left(x_{A}^{i}+x_{B}^{i}=2\right)=\frac{1}{4}.$$

The posterior probability of each case is

(26) $$\begin{array}{cc} P\left(x_{A}^{i}+x_{B}^{i}\right.\left.=0|y_{R}^{i}\right) & =\frac{P\left(x_{A}^{i}+x_{B}^{i}=0\right)P\left(y_{R}^{i}|x_{A}^{i}+x_{B}^{i}=0\right)}{P\left(y_{R}^{i}\right)}\\ \; & =\frac{1}{4\sqrt{2\pi }\sigma P\left(y_{R}^{i}\right)}\exp\left(-\frac{{\left(y_{R}^{i}\right)}^{2}}{2\sigma^{2}}\right), \end{array}$$

(27) $$\begin{array}{cc} P\left(x_{A}^{i}+x_{B}^{i}\right.\left.=1|y_{R}^{i}\right) & =\frac{P\left(x_{A}^{i}+x_{B}^{i}=1\right)P\left(y_{R}^{i}|x_{A}^{i}+x_{B}^{i}=1\right)}{P\left(y_{R}^{i}\right)}\\ \; & =\frac{1}{2\sqrt{2\pi }\sigma P\left(y_{R}^{i}\right)}\exp\left(-\frac{{\left(y_{R}^{i}-h\right)}^{2}}{2\sigma^{2}}\right), \end{array}$$

(28) $$\begin{array}{cc} P\left(x_{A}^{i}+x_{B}^{i}\right.\left.=2|y_{R}^{i}\right) & =\frac{P\left(x_{A}^{i}+x_{B}^{i}=2\right)P\left(y_{R}^{i}|x_{A}^{i}+x_{B}^{i}=2\right)}{P\left(y_{R}^{i}\right)}\\ \; & =\frac{1}{4\sqrt{2\pi }\sigma P\left(y_{R}^{i}\right)}\exp\left(-\frac{{\left(y_{R}^{i}-2h\right)}^{2}}{2\sigma^{2}}\right). \end{array}$$

The LLR of $y_{R}$ can be further simplified as

(29) $$\begin{array}{cc} L\left(y_{R}^{i}\right) & =\ln\frac{P\left(x_{A}^{i}+x_{B}^{i}=0|y_{R}^{i}\right)+P\left(x_{A}^{i}+x_{B}^{i}=2|y_{R}^{i}\right)}{P\left(x_{A}^{i}+x_{B}^{i}=1|y_{R}^{i}\right)}\\ \; & =\ln\frac{\exp\left(-\frac{{\left(y_{R}^{i}\right)}^{2}}{2\sigma^{2}}\right)+\exp\left(-\frac{{\left(y_{R}^{i}-2h\right)}^{2}}{2\sigma^{2}}\right)}{2\exp\left(-\frac{{\left(y_{R}^{i}-h\right)}^{2}}{2\sigma^{2}}\right)}. \end{array}$$

Based on the computed results, the relay node performs a specific mapping operation to obtain $u_{R}$. This $u_{R}$ is then encoded using a polar encoder to produce $c_{R}$. After modulation, the resulting signal $x_{R}$ is transmitted to both A and B;

*(3).* Nodes A and B receive and decode the signal sent by the relay individually. For node A, the received signal can be expressed as $y_{A}=hx_{R}+n_{A}$, where $n_{A}$ represents the noise introduced during transmission. The LLR for $y_{A}$ is calculated as

(30) $$\begin{array}{cc} L\left(y_{A}^{i}\right) & =\ln\frac{P\left(c_{R}^{i}=0|y_{R}^{i}\right)}{P\left(c_{R}^{i}=1|y_{R}^{i}\right)}\\ \; & =\ln\frac{\exp\left(-\frac{{\left(y_{A}^{i}\right)}^{2}}{2\sigma^{2}}\right)}{\exp\left(-\frac{{\left(y_{A}^{i}-h\right)}^{2}}{2\sigma^{2}}\right)}\\ \; & =\frac{{\left(y_{A}^{i}-h\right)}^{2}-{\left(y_{A}^{i}\right)}^{2}}{2\sigma^{2}}, \end{array}$$

where $y_{A}^{i}\in y_{A}$. The calculation method at node B is similar. ${\hat{u}}_{R}$ can be decoded using the LLR of $y_{A}$.

Node A can recover the information from node B by XORing its original information with ${\hat{u}}_{R}$, resulting in ${\hat{u}}_{B}={\hat{u}}_{R}⊕u_{A}$. Similarly, node B can retrieve the information sent by node A, thereby completing the information exchange between the two nodes. Compared to the previous scheme, our approach requires just one decoding operation at the relay node, reducing computational complexity by almost 50%. Furthermore, with only one set of equipment needed for information exchange, device costs are also reduced by 50%.

## 4. Simulation Results

To validate the VLC system model, we simulate and analyze the illumination conditions and luminous power on the plane where the receiver is positioned. In this investigation, the distance between the receiving plane and the light source is set at 2.15 m.

**Figure 5:**
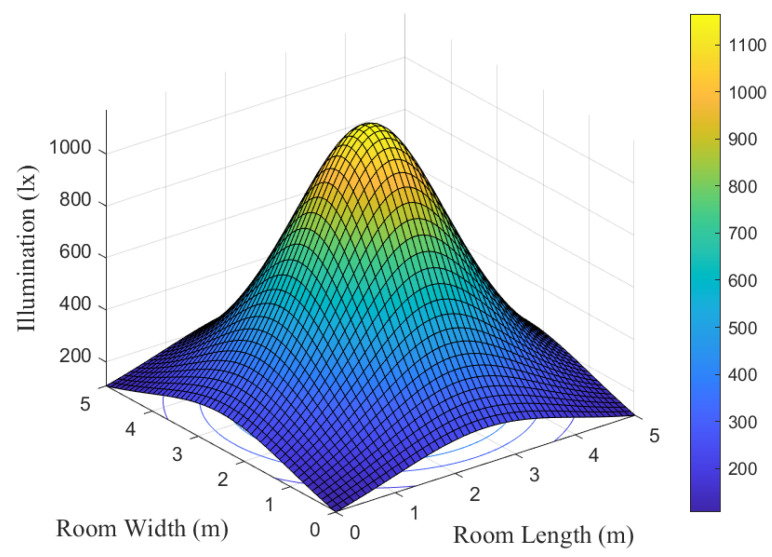
The illumination distribution diagram.

**Figure 6:**
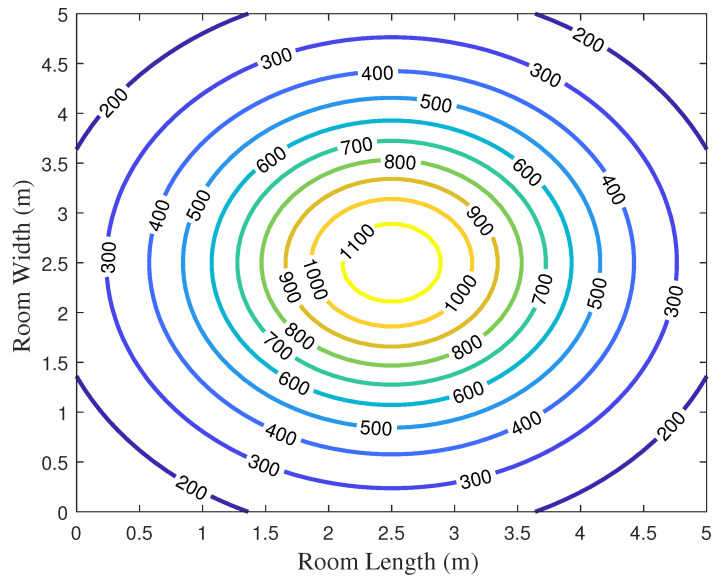
The contour map of illumination.

Figure 5 and Figure 6 show the illumination condition of the indoor VLC system. It is evident that the area near the light source experiences higher illumination levels. From the center towards the periphery, the illumination intensity gradually diminishes. Overall, the illumination ranges between 300 lux and 1100 lux, aligning with the regulations of the International Organization for Standardization and adequately meeting practical lighting requirements.

**Figure 7:**
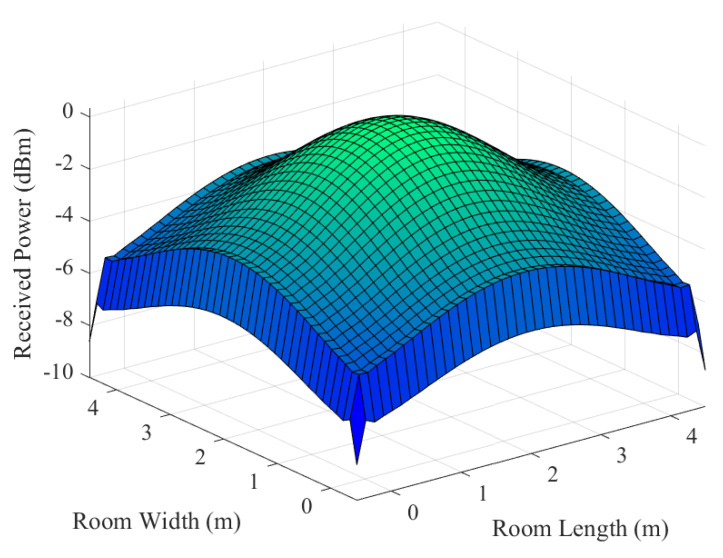
The luminous power distribution diagram.

**Figure 8:**
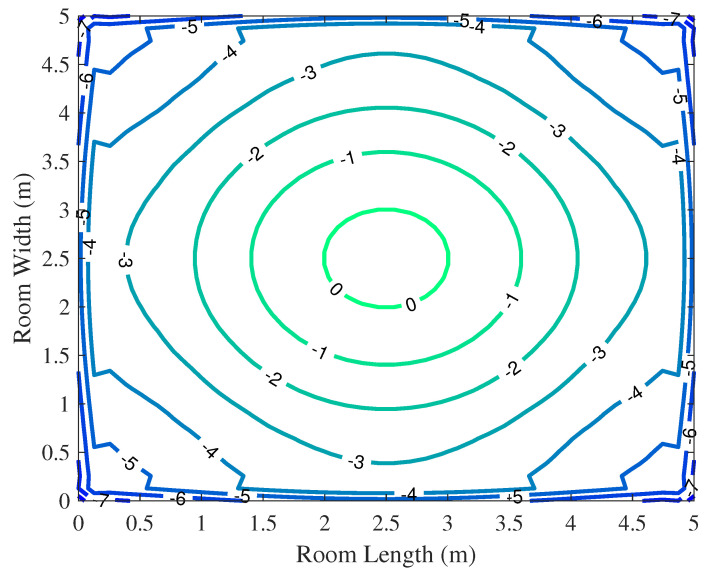
The contour map of luminous power.

Figure 7 and Figure 8 show the luminous power of the model described. The received power ranges from −5 dBm to 0 dBm. Notably, the maximum received power is directly beneath the LEDs, with a gradual decrease towards the surroundings, which is basically consistent with Lambert’s law. This analysis affirms the validity of the model’s layout, effectively striking a balance between communication and illumination. Thus, the constructed model stands as an accurate and rational representation.

**Figure 9:**
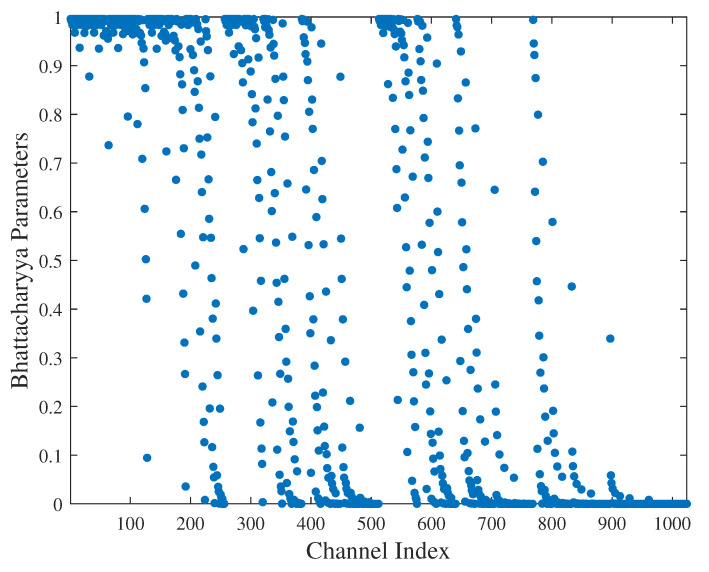
The distribution diagram of Bhattacharyya parameter.

Subsequently, we delve into the technical advantages offered by the proposed scheme by employing polar codes for VLC. In our simulations, we use a polar code with a length of 1024 and a code rate of 0.5. The Bhattacharyya parameters of the polarized channels within the VLC model are calculated using Equation (12), and the scatter plot is displayed in Figure 9. The x-axis represents the indices of the polarized channels, while the y-axis shows the values of the Bhattacharyya parameters $Z(W_{N}^{\left(i\right)})$. By examining the relationship between the Bhattacharyya parameters and channel symmetric capacity, a significant polarization phenomenon is observed. Specifically, some channels’ symmetric capacities trend towards 0, while others approach 1, indicating that the channel reliability estimation is ideal.

**Figure 10:**
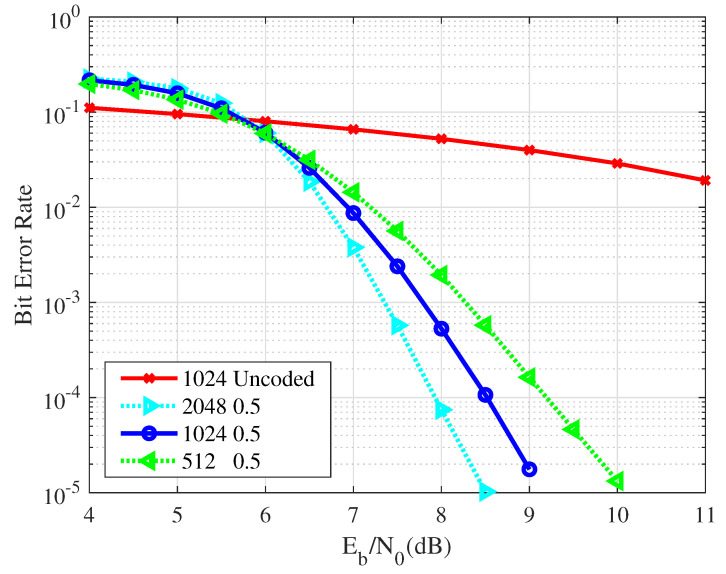
A comparison of the BER performance for the VLC system with a code rate of 0.5 and code lengths of 512, 1024, and 2048.

Figure 10 illustrates the BER of the VLC system. In this analysis, polar codes with a code rate of 0.5 and different code lengths are used in the simulations. When the SNR exceeds a certain threshold, it becomes clear that the BER curve for the coded VLC system consistently outperforms that of the uncoded scenario, with a noticeable gap between the two. Furthermore, when the code rate remains fixed, the error-resistance performance of the polar codes improves with increasing code length. This enhancement is due to the stronger polarization effect observed in the sub-channels as the code length increases. Thus, it is evident that the use of polar codes significantly enhances the communication quality of the VLC system.

**Figure 11:**
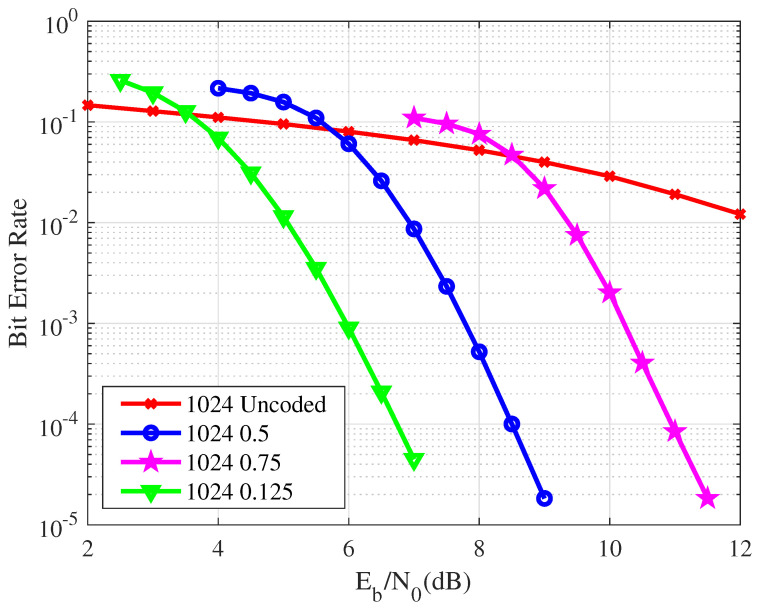
A comparison of the BER performance for the VLC system with a code length of 1024 and code rates of 0.125, 0.5, and 0.75.

Figure 11 illustrates the BER of the VLC system utilizing polar codes with a code length of 1024 and code rates of 0.75, 0.5, and 0.125. At a BER of $10^{-2}$, the proposed scheme achieves coding gains of approximately 2.5 dB, 5 dB, and 7 dB, compared to the uncoded scenario across the different code rates. In summary, when the code length remains constant, lower code rates exhibit superior error correction capabilities, while higher code rates are comparatively less effective. With respect to different code rates, distinct ranges of SNR are required to facilitate reliable communication in the VLC system. Telecommunication performance may decline if the SNR falls below a certain threshold.

**Figure 12:**
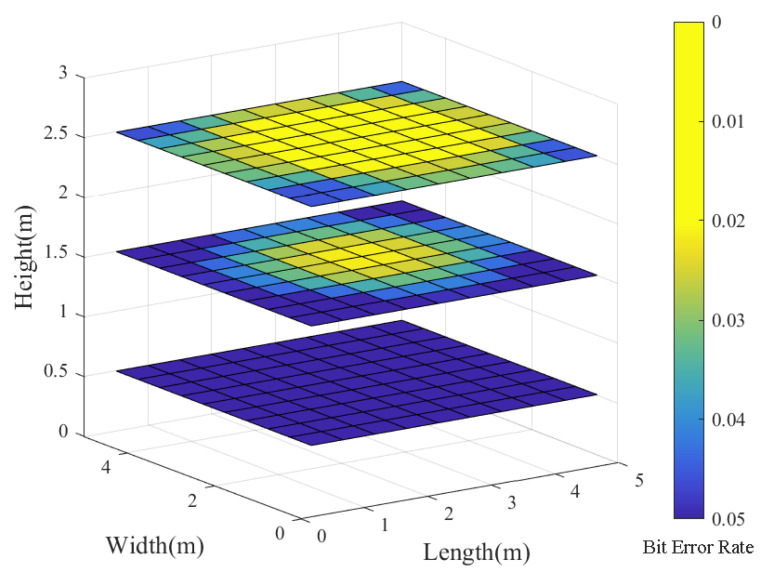
The BER slice figure of the uncoded VLC system at varying heights—specifically, at 0.5 m, 1.5 m, and 2.5 m.

**Figure 13:**
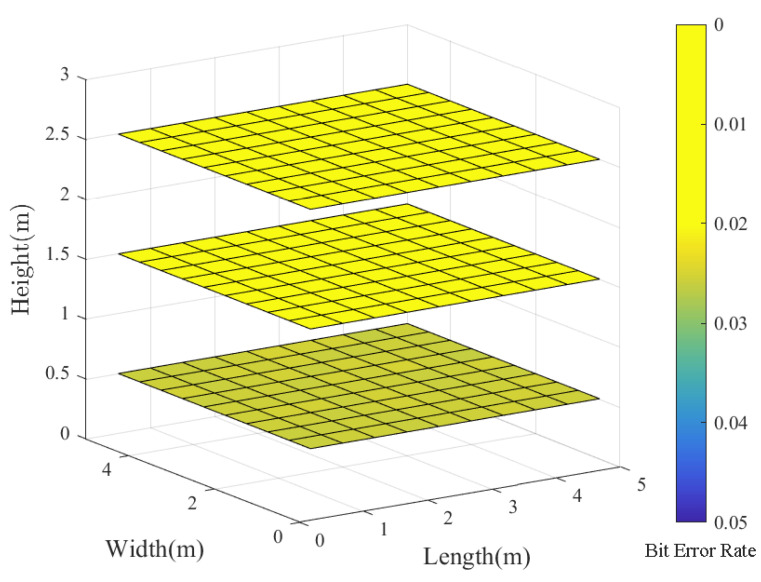
The BER slice figure of the coded VLC system at varying heights—specifically, at 0.5 m, 1.5 m, and 2.5 m.

With practical VLC applications in mind, we then examine the BER distribution in an indoor VLC system at various communication heights. Our experimentation involves polar codes with a code length of 1024 and a code rate of 0.5, comparing the results to an uncoded system. Figure 12 and Figure 13 illustrate the following:

*(1).* As the communication distance increases, both coded and uncoded VLC systems experience an increase in BER. This is due to higher noise levels in the VLC channel over longer distances, resulting in a decline in transmission quality;

*(2).* When tested at different heights within the room, the VLC system that utilizes polar codes demonstrates a substantially lower BER compared to an uncoded system. Overall, the improvement in BER becomes more significant as communication distances increase. Thus, we conclude that polar codes have the potential to extend the range of reliable communication and significantly boost the performance of the VLC system.

**Figure 14:**
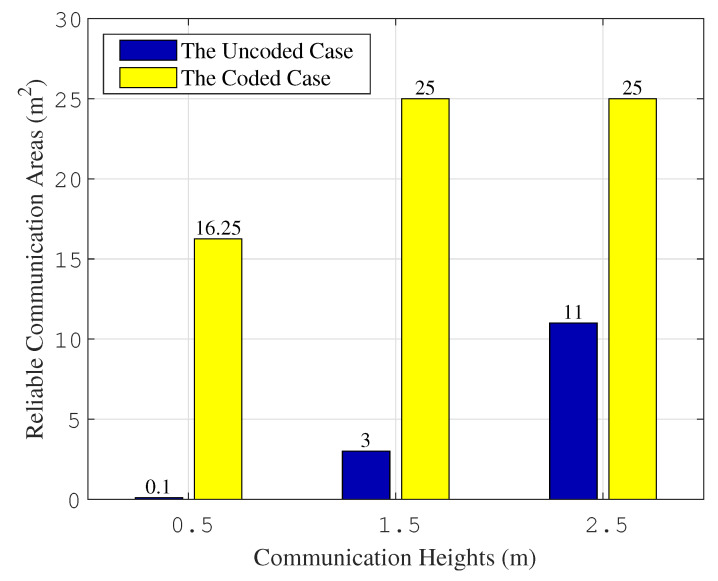
The statistical results for reliable communication area.

Figure 14 presents the calculated results for the reliable communication area of the VLC system at various communication heights. It is clear that implementing the polar code scheme examined in this study significantly expands the reliable communication area of the VLC system, especially at communication heights of 1.5 m and 2.5 m, where it encompasses the entire room. Thus, the proposed scheme notably bolsters the communication reliability of the VLC system.

**Figure 15:**
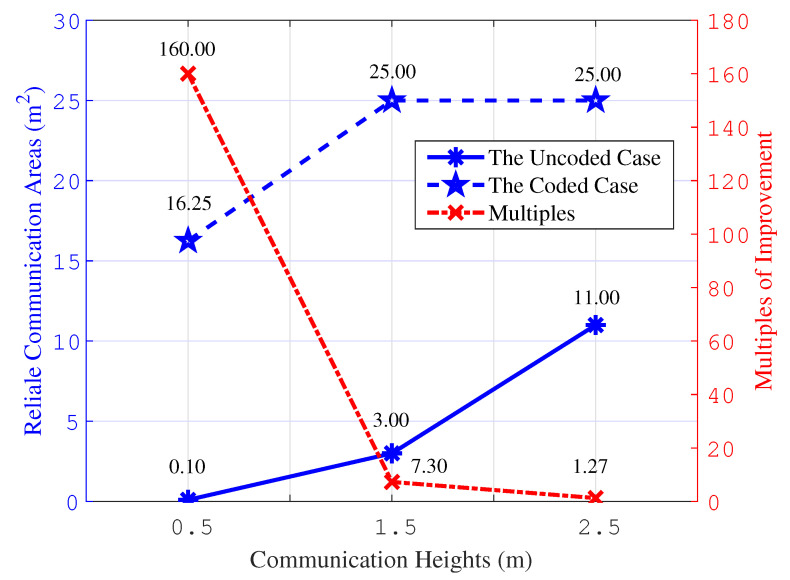
The improvement effects for reliable communication area.

Figure 15 shows the improvement effects of the studied scheme on the reliable communication area of the VLC system. At a communication height of 0.5 m, the BER of the VLC system exceeds 10^−3^, indicating a reliable communication area of 0. For the ease of calculation, 0.1 is taken as a representative value. From the simulation outcomes, it is evident that the reliable communication area, for both encoded and uncoded scenarios, steadily increases with the rise in communication heights. As the communication distance extends, the improvement brought by the proposed scheme becomes more pronounced. This implies that the expansion effect on the effective communication range is more significant for greater communication distances. The calculation results affirm the effectiveness of the studied encoding scheme.

We then evaluate the BER performance of the collaborative coding schemes introduced earlier. In the simulation setup, A and B each encode 512 information bits using a polar encoder with a code rate of 1/2, producing coded words of length 1024. For the DNC-Polar relay scheme, the network-coded results are treated as the information bits and are also encoded using the polar coding with a code rate of 1/2. In contrast, for the PNC-Polar relay scheme, the encoded results from the mapped superimposed VLC signals are used as input for the encoding process.

The results of the simulations demonstrate that, in comparison to cooperative coding relay schemes, the conventional relay approach exhibits a lower BER. Traditionally, the relay node independently decodes the optical signals received from the source nodes, with the aim of correcting errors. Following this decoding process, the relay forwards the corrected signals to the destination nodes, thereby effectively minimizing errors and achieving greater signal gain. However, this scheme processes the VLC signals individually at the relay node, causing a significant transmission delay. As a result, it may not be ideal for wireless communication systems. In contrast, cooperative coding schemes reduce the number of time slots required, potentially improving the throughput rate of the VLC system.

**Figure 16:**
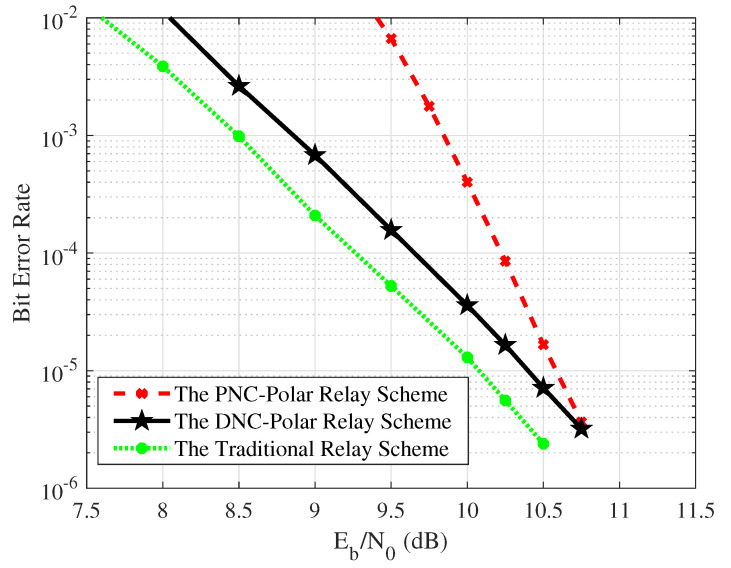
The BER performance of different relay schemes in a VLC system.

As shown in Figure 16, in low-SNR scenarios, a noticeable performance gap is observed between the DNC-Polar and PNC-Polar relay schemes. Specifically, at a BER of $10^{-3}$, the PNC-Polar scheme exhibits a performance gap of approximately 1 dB. However, the BER curve for the PNC-Polar relay scheme shows a steeper decline. As the SNR increases, the performance gap between the two schemes gradually decreases, and, at an SNR of 10.75 dB, the BERs of both relay schemes become nearly identical. This suggests that, in high SNR conditions, the two schemes can achieve comparable telecommunication performance.

In comparison to the PNC-Polar relay scheme, the DNC-Polar relay scheme exhibits superior performance in terms of BER. This is because, in the PNC-Polar scheme, the relay node relies on superimposed signals, which are affected by noise, to compute soft information, potentially leading to inaccuracies. Consequently, errors may arise during multiple decoding operations, degrading the quality of bidirectional communication. In contrast, the DNC-Polar relay scheme ensures that signals from source nodes are processed independently, eliminating the potential for interference caused by signal superposition. Each signal is decoded individually, resulting in fewer errors and enhancing the reliability of the VLC system.

In terms of transmission efficiency and cost-effectiveness, the PNC-Polar relay scheme outperforms the DNC-Polar relay scheme. Specifically, it necessitates only two time slots and a single set of receiving equipment at the relay node, as opposed to the three time slots and two sets of equipment required by the DNC-Polar scheme. This advantage of the PNC-Polar scheme significantly enhances the VLC system’s transmission efficiency and minimizes equipment costs. Furthermore, at high SNR levels, the PNC-Polar relay scheme demonstrates comparable telecommunication performance with reduced operational complexity. The simulated BER outcomes validate the practicality and effectiveness of this innovative scheme.

**Figure 17:**
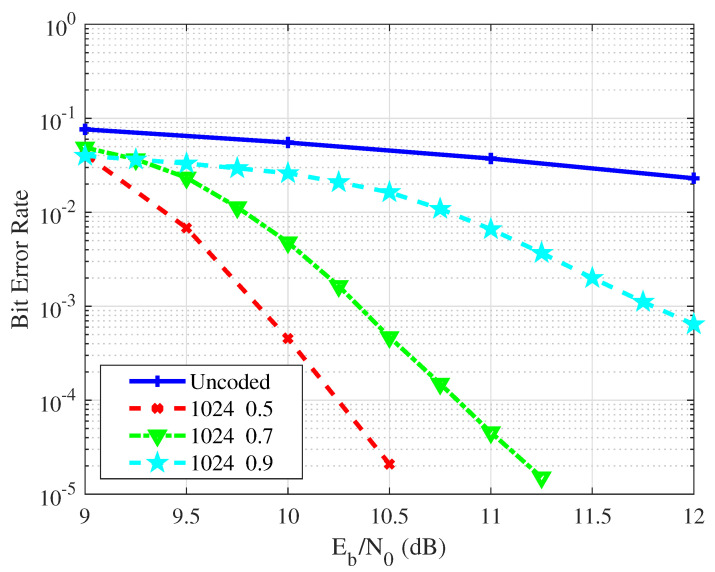
The BER performance of the proposed scheme across different code rates.

Figure 17 shows the BER results of the uncoded case and the joint design scheme of polar code and PNC in the VLC relay system. In these simulations, the code length is set to 1024, and the code rates are 0.5, 0.7, and 0.9. As shown in the results, the BER curve of the joint design scheme is significantly lower than that of the uncoded case. The proposed scheme effectively corrects erroneous data generated in telecommunication and improves the reliability of visible light signals. At a BER of $10^{-2}$, a coding gain of approximately 3 dB is observed between the uncoded case and the 0.5 code rate condition. This performance gain is expected to increase with higher SNR levels. At the same time, the relay node adopts the specific mapping to process the superimposed signals directly, converting the mutual interference between them into valuable information that aids in the completion of telecommunications. This leads to a reduction in transmission time slots and an increase in network throughput capacity. The proposed scheme’s efficacy and feasibility are thus confirmed.

**Figure 18:**
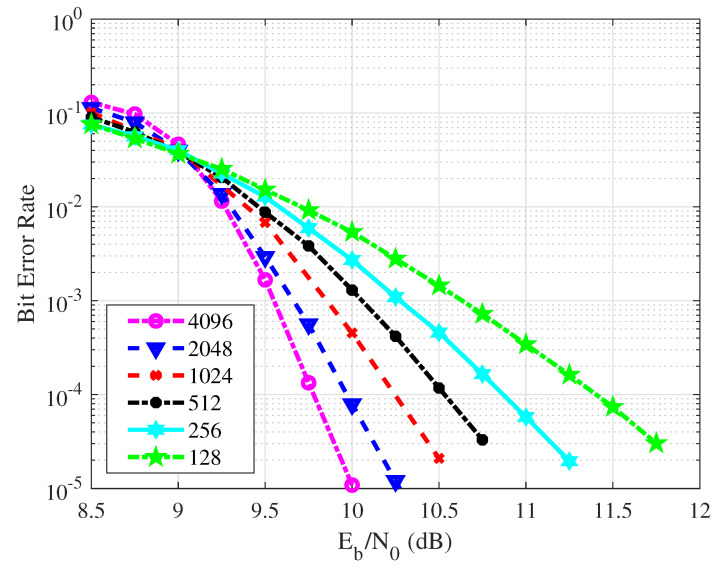
The BER performance of the proposed scheme with code lengths.

Figure 18 illustrates the BER results of the VLC relay system, adopting the proposed scheme with varying code lengths. The experiments cover code lengths ranging from 128 to 4096, all at a fixed code rate of 0.5. Generally, as the code length increases, a more pronounced downward trend in the BER curve is observed. When the BER exceeds $10^{-2}$, the performance distinctions among different code lengths are relatively minor. However, for BERs lower than $10^{-2}$, code length variations have a notable impact on the error control capability of the VLC system. Therefore, in real-world communication scenarios, it is crucial to consider actual requirements and select an appropriate code length. It is worth noting that excessively long codes may lead to increased decoding latency, potentially affecting communication timeliness.

**Figure 19:**
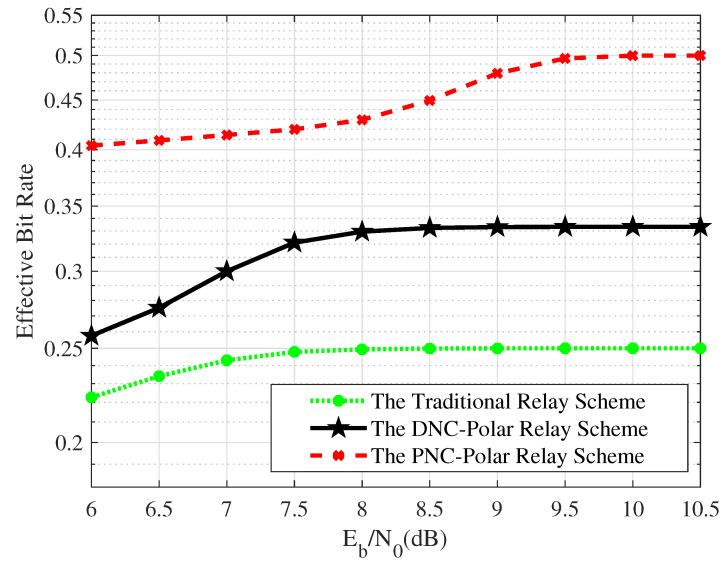
The effective bit rate per unit time-slot of relay schemes.

Figure 19 shows the effective bit rates per unit time-slot of the VLC system that adopts different relay schemes. The effective bit rates per unit time-slot of the three relay schemes gradually increase with the growth of SNR. The main reason is that the amount of correctly transmitted information increases when the condition of the VLC channel is optimal. The effective bit rates of the PNC-Polar scheme are significantly higher than the other two relay schemes. When the SNR surpasses 9 dB, the traditional relay scheme stabilizes at an efficiency of 0.25, while the DNC relay scheme reaches 0.33, and the PNC relay scheme achieves 0.5. These correspond to 100% and 51.5% increasse in transmission efficiency for the PNC and DNC schemes, respectively.

**Figure 20:**
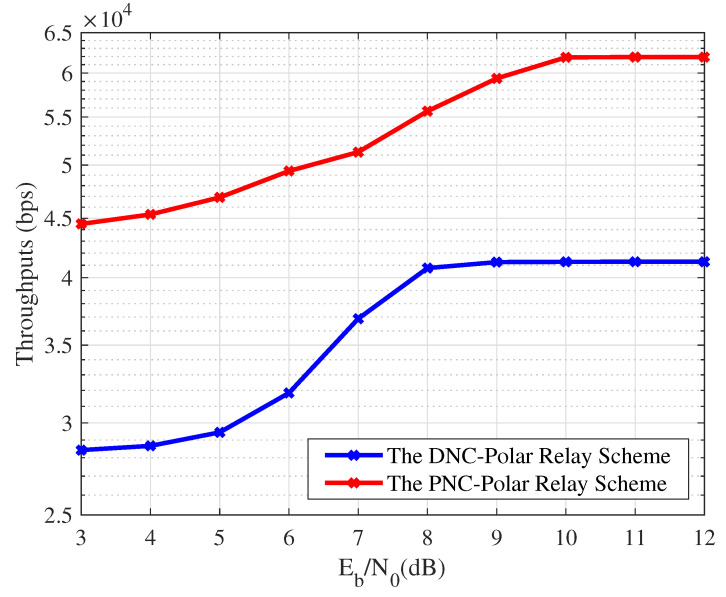
The throughput comparision of relay schemes.

Figure 20 shows the throughput of the VLC system, employing different collaborative coding schemes. In the experiments, the length of the uncoded sequence is 512, the code rate is 0.5, and the source generates $10^{3}$ frames of data per second. The correct amount of data transmitted within a certain period of time is counted to calculate the throughput. Overall, the throughput of the designed scheme is 1.5 times higher compared to the DNC-Polar relay scheme. When the SNR is higher than 10 dB, the BERs of the two schemes are close to each other, but the information exchange rate in the PNC-Polar relay scheme is significantly improved by 50%, which balances the transmission reliability and effectiveness in the VLC system.

## 5. Conclusions

In this study, we present a construction framework for polar codes that is specifically designed for VLC channels. Simulation results showed that the proposed VLC system, utilizing these codes, achieved a coding gain of at least 5 dB. Adjusting the polar code parameters further reduced the system’s BER. Notably, across various indoor heights, the BER of the VLC system using our approach was significantly lower than that of an uncoded system, with this advantage becoming more pronounced as communication distances increased. Our scheme effectively reduced transmission errors in the VLC system, greatly extending the range for reliable communication. We also introduced a mapping strategy that combined polar codes with PNC in the VLC system. The results revealed distinctive performance characteristics between the proposed methodology and conventional transmission strategies. At a BER of $10^{-5}$, this performance differential was small, yet our approach conserved two time slots and effectively doubled the throughput. In contrast to the DNC-Polar relay method, our approach saved one time slot, boosting throughput by half. Furthermore, it necessitated only a single receiving equipment set at the relay node, reducing equipment costs by half. Overall, the proposed framework enhanced VLC system throughput, improved telecommunication quality, and was well-suited for low-latency VLC systems.

## Figures and Tables

**Table 1:** The relay mapping rule.

$c_{A}$	$c_{B}$	$x_{A}$	$x_{B}$	$y_{R}$	$c_{A}⊕c_{B}$
0	0	0	0	0	0
0	1	0	1$\left(P_{t}\right)$	1 $\left(P_{r}\right)$	1
1	0	1$\left(P_{t}\right)$	0	1 $\left(P_{r}\right)$	1
1	1	1$\left(P_{t}\right)$	1$\left(P_{t}\right)$	2$\left(2P_{r}\right)$	0

## Data Availability

Data are available from the authors, on request.

## Abbreviations

Below is a list of abbreviations used throughout this paper, along with their corresponding definitions.

BEC	Binary Erasure Channels
BER	Bit Error Rate
DNC	Direct Network Coding
FEC	Forward Error Correction
FL	Federated Learning
GA	Gaussian Approximation
GMAC	Gaussian Multiple-Access Channels
IM/DD	Intensity Modulation Direct Detection
LEDs	Light-Emitting Diodes
LiSAC	Lighting, Sensing, and Communication
LLR	Log-Likelihood Ratio
LOS	Line of Sight
NLOS	Non-Line of Sight
NOMA	Non-Orthogonal Multiple Access
OFDM	Orthogonal Frequency Division Multiplexing
OOK	On–Off Keying
PNC	Physical-Layer Network Coding
RF	Radio Frequency
SNR	Signal-to-Noise Ratio
VLC	Visible Light Communication
VLP	Visible Light Positioning
